# Posterior nutcracker syndrome: a case report

**DOI:** 10.1186/s13256-020-02617-0

**Published:** 2021-02-01

**Authors:** Cecilia Gozzo, Renato Farina, Pietro Valerio Foti, Francesco Aldo Iannace, Andrea Conti, Isabella Pennisi, Serafino Santonocito, Stefano Palmucci, Antonio Basile

**Affiliations:** Radiodiagnostic and Radiotherapy Unit, Department of Medical and Surgical Sciences and Advanced Technologies “GF Ingrassia”, Via Santa Sofia 78, 95123 Catania, Italy

**Keywords:** Nutcracker syndrome, Doppler ultrasound, Cardiovascular abnormalities, Multidetector computed tomography

## Abstract

**Background:**

Posterior nutcracker syndrome is defined by the compression of the left renal vein between the abdominal aorta and a lumbar vertebral body. It can be clinically manifest with intermittent hematuria, gonadal or spermatic reflux resulting in varicocele. Ultrasound is the first-line imaging which require  more accurate study  with contrast-enhanced computed tomography. Management can be conservative in younger patients with mild hematuria due to the high spontaneous remission rate and invasive with open surgical and endovascular interventions. We describe a very rare case with compression of the left renal vein due to an osteophyte of the spine.

**Case presentation:**

A 62-year-old Caucasic male came to our radiology department for chronic hepatitis B virus (HBV)-related liver disease follow-up and mild scrotal pain. The ultrasound examination revealed a compression of the left retro-aortic renal vein in the aorto-vertebral space caused by an osteophyte. Duplex Doppler ultrasound revealed flow congestion in the left renal vein and renal failure; power Doppler ultrasound showed left varicocele.

**Conclusions:**

Doppler ultrasound is the first-line imaging and allows the detection of all the typical signs of posterior nutcracker: left renal vein stenosis, flow congestion and renal failure. Nutcracker syndrome should be suspected in older patients with left varicocele associated with hematuria. Failure to diagnose and treat these patients could have serious consequences for their health.

## Introduction

**Fig. 1:**
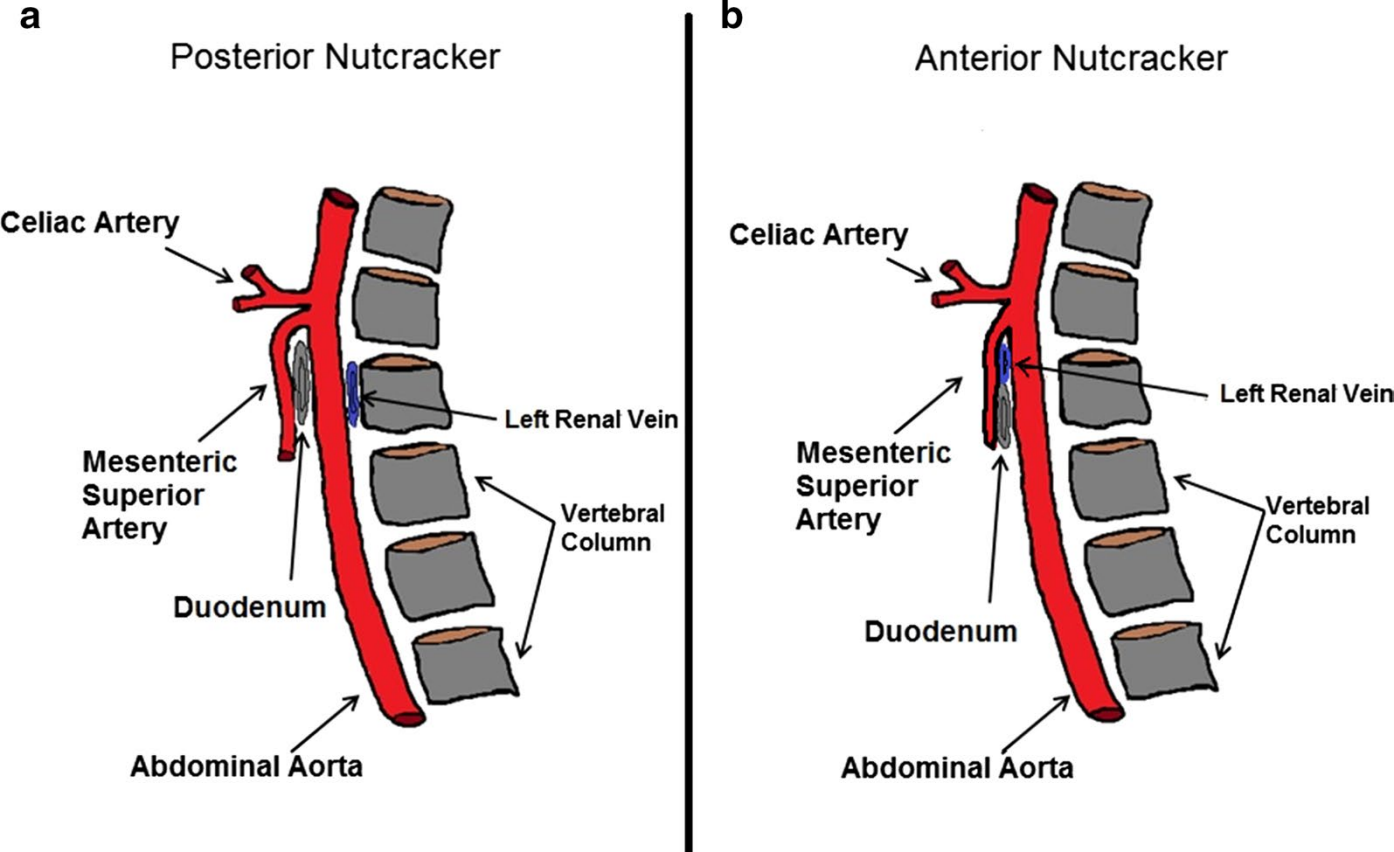
**a** Diagram showing the anatomical relationships between the left renal vein and the vertebral column in posterior nutcracker syndrome. **b** Diagram showing the anatomical relationships between the left renal vein and the aorto-mesenteric space in anterior nutcracker syndrome

Nutcracker syndrome (NCS) belongs to a group of very rare vascular disorders known as vascular compression syndromes, the most well known of which are May-Thurner [1], Dunbar [2] and thoracic outlet syndrome [3]. NCS is caused by compression of the left renal vein (LRV) and can be congenital or acquired. There are two types: anterior (ANCS) and posterior nutcracker syndrome (PNCS); in the first, compression of the LRV occurs because of a congenital or acquired reduction of the aorto-mesenteric space inside which the LRV passes, in particular when the distance between the superior mesenteric artery and the abdominal aorta is < 8 mm and the aorto-mesenteric angle compression < 22°; it can be combined with Wilkie's syndrome, in which the duodenum is compressed simultaneously (Fig. 1a, b) [4]. PNCS, also known as “pseudo-nutcracker syndrome,” is defined by the compression of the LRV between the abdominal aorta and a lumbar vertebral body [5, 6]. PNCS can be caused by an underlying anatomical variant (retro-aortic LRV or renal collar) or aorta and vertebral pathology (abdominal aneurysms or lumbar osteophytosis) [7–9]. PNCS can clinically manifest with intermittent hematuria, gonadal or spermatic reflux and pelvic varices or only be seen on imaging without clinical manifestation in the so-called “nutcracker phenomenon” [10, 11]. The retro-aortic LRV is responsible for this syndrome in 9% of patients with left-sided varicocele [7]. The “renal collar,” also known as the “circum-aortic venous ring,” is another anatomic variant characterized by the presence of two LRVs passing anteriorly and posteriorly to the aorta, respectively [7]. In the “renal collar” condition, the concomitant compression of the anterior LRV (between the abdominal aorta and superior mesenteric artery) and the posterior LRV (between the abdominal aorta and spine) results in “combined nutcracker syndrome" [12]. Doppler ultrasound is the first-line imaging which allows detection of left renal vein stenosis, possible renal failure and varicocele secondary to venous congestion in the LRV, which require more accurate study with contrast-enhanced computed tomography (CE-CT) [10, 12, 13]. A CE-CT scan provides accurate detection of vascular structures and their relationship to adjacent organs and allows excluding other causes of compression [11]. Management of NCS can be both conservative and invasive, depending on the grade of hematuria and pain [14, 15]. PNCS treatment can be conservative in younger patients with mild hematuria because of the high spontaneous remission rate [14]. Invasive treatments such as open surgical and endovascular interventions are proposed in case of recurrent gross hematuria, severe flank pain or ineffective conservative treatment [12, 15]. Surgical treatment of PNCS consists of anterior reimplantation of the retroaortic LRV into the inferior vena cava [14]. Endovascular LRV stenting may be considered in case of associated pelvic congestion [12, 16]. We describe a very rare case with compression of the left renal vein due to an osteophyte of the spine.

## Case presentation

A 62-year-old Caucasic male came to our radiology department for chronic hepatitis B virus (HBV-)related liver disease follow-up. He weighed 54 kg and had a body mass index of 21.35. He also complained mild scrotal pain without any others symptoms.

**Fig. 2:**
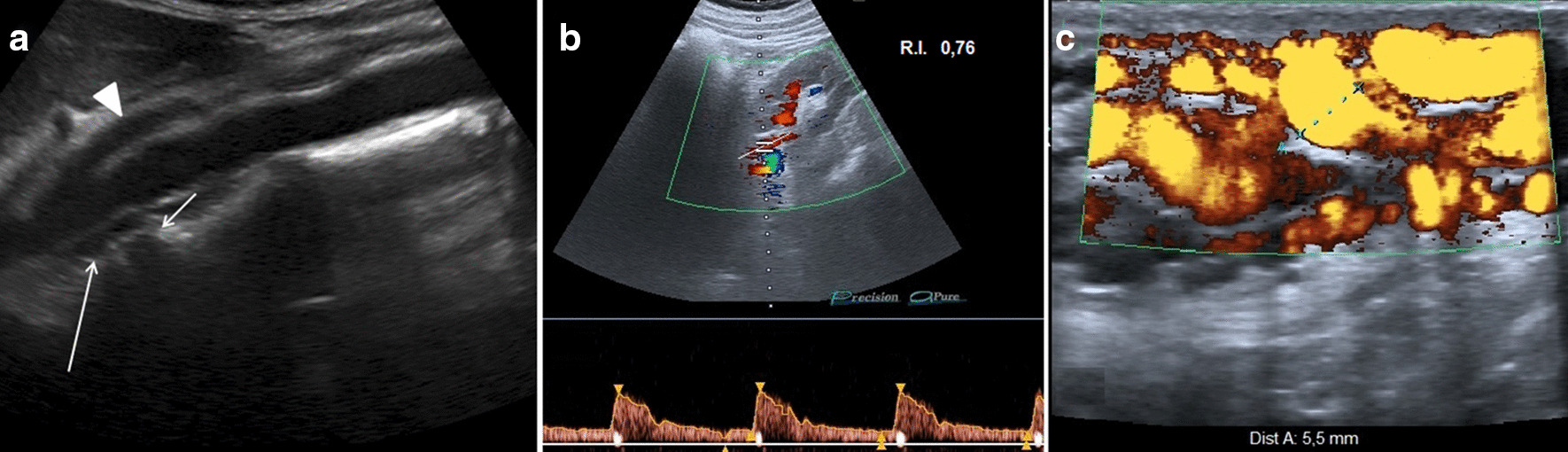
**a** B-mode US: longitudinal sub-xyphoid scan of the abdominal aorta at the level of the arising superior mesenteric artery (arrowhead), showing the retro-aortic left renal vein (long arrow) compressed between the abdominal aorta (*) and an osteophyte (short arrow) of the third lumbar vertebral body. **b** Duplex Doppler US of the left renal artery showing a higher resistive index value (0.76). **c** Power Doppler US of the pampiniform plexus showing left varicocele (grade IV on Sarteschi’s scale)

**Fig. 3:**
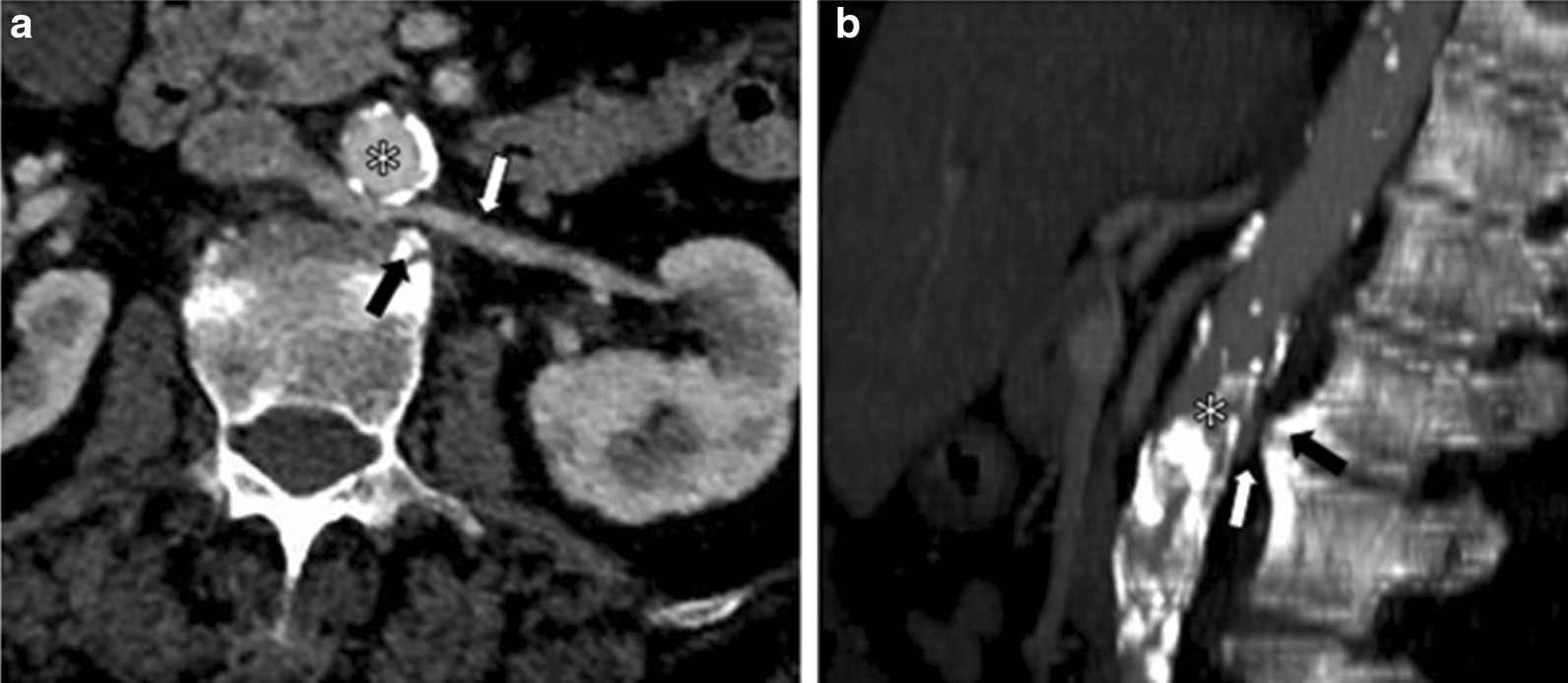
Multidetector CT angiography. **a** Axial reconstruction and **b** sagittal reconstruction show the retro-aortic left renal vein (white arrow), which is compressed between the abdominal aorta (*) and osteophyte (black arrow) of the third lumbar vertebral body

**Fig. 4:**
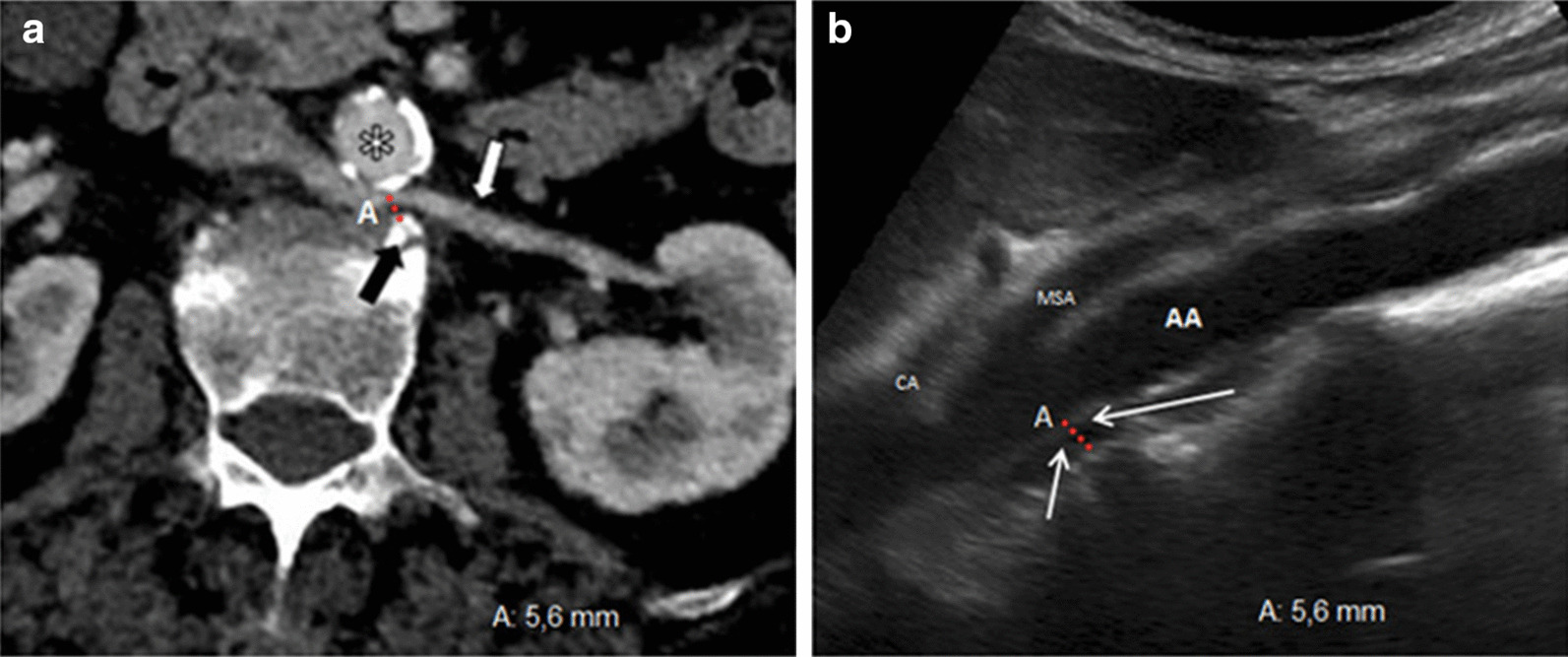
Aorto-vertebral space. **a** Measurement of the aorto-vertebral distance (A) with multi-detector computed tomography. Retro-aortic left renal vein (white arrow). Abdominal aorta (*). Osteophyte (black arrow) of the third lumbar vertebral body. **b** Measurement of the aorto-vertebral distance (A) with B-mode ultrasound. Left renal vein (short arrow). Aorto-vertebral space (long arrow). AA = abdominal aorta; MSA = mesenteric superior artery; CA = celiac artery

The patient underwent ultrasound and multidetector computed tomography (MDCT) of the abdomen. The ultrasound examination was performed with an Aplio XG (Toshiba) device with 7.5-MHz linear and 3.5-MHz convex probes. Ultrasound scans included B-mode ultrasound (B-mode US), color Doppler ultrasound (color Doppler US), power Doppler ultrasound (power Doppler US) and duplex Doppler ultrasound (duplex Doppler US) techniques. Color Doppler US showed compression of the retro-aortic LRV in the aorto-vertebral space (Fig. 2a). Duplex Doppler US showed a reduction of the peak speed velocity (PSV) of the pre-stenotic tract and an increase of the PSV in the post-stenotic tract, flow ratio (FR) (PSV post-stenotic/PSV pre-stenotic) > 2.5, resistive index (RI) of the left renal artery above the norm (0.76) (Fig. 2b) and regular flow and RI in the right renal artery (used as a control). Power Doppler US of the pampiniform plexus revealed left varicocele (Sarteschi's IV degree) (Fig. 2c); the results are summarized in Table 1. The patient underwent an MDCT angiography examination with a multi-detector CT (Optima 64 slice, GE Healthcare). The CT scan showed a retro-aortic LRV compressed between the abdominal aorta and an osteophyte of the third lumbar vertebral body (Fig. 3a, b). The distance between the abdominal aorta and the spine measured by ultrasound and MDCT was 5.6 mm (Fig. 4a, b). The ultrasound was performed by an operator with 20 years of experience.

**Table 1:** Summary of the results

	PSV	Pre-stenotic PSV	Post-stenotic PSV	Flow ratio	Minimum distances between the abdominal aorta and vertebral soma	Resistive index (RI)
LRV		22.3 cm/s	56.4 cm/s	2.52		
RRV	28.8 cm/s					
Aorto-vertebral space					5.6 mm	
Left kidney						0.76
Right kidney						0.62

*PSV* peak speed velocity.; *LRV *left renal vein; *RRV* right renal vein

## Discussion

NCS can be recognized by correlating imaging findings to the clinical presentation. The difficulty of diagnosis lies in the aspecifity of symptoms that can be also caused by other diseases. More challenging is the diagnosis of the nutcracker phenomenon due to the lack of symptoms. In fact, this latter is often an occasional detection during routine imaging studies. Retro-aortic LRV compression (PNCS) is more rare than ANCS. PNCS can be related to the compression of the retro-aortic LRV between the abdominal aorta and lumbar vertebral osteophytosis. In our opinion, PNCS must be suspected in older patients with left varicocele associated with hematuria, and imaging examinations are crucial to formulate the diagnosis.

Therefore, on the basis of the ultrasound findings, we were able to obtain a complete diagnostic estimate of the disease; in addition, determination of the flow ratio gave us an estimate of the degree of stenosis, which was important for the subsequent therapeutic planning. An FR > 2.5 cm/s corresponds to a > 50% stenosis [17]; this latter  is considered hemodynamically significant if >70%. The narrowed aorto-vertebral space (5.6 mm) did not provide, in our case, sufficient guarantees of the procedure's success and long-term endovascular stenting [18], while surgery, which is much more invasive than the interventional technique, was rejected by the patient. Considering FR and RI values  (respectively just over 2.5 and 0.76), we decided to maintain a conservative therapeutic approach by  monitoring renal functional parameters (creatininemia, glomerular filtrate) and the flow of the LRV with color and duplex Doppler US with a 6-month interval.

The presence of vertebral osteophytosis requires long-term follow-up of the LRV in order to detect increase of stenosis and complications (i.e. thrombosis).

MDCT confirmed the ultrasound findings and ruled out other causes of LRV stenosis, but, unlike ultrasound, it could not provide information on the flow and degree of stenosis, which, in our case, was valuable for therapeutic planning. Especially for ANCS, the use of MDCT is of greater importance when it is necessary to establish whether compression of the LRV is associated with compression of the duodenum.

Unlike ANCS, there is still no aorto-vertebral distance cut-off for PNCS, and establishing the limit value below which the syndrome occurs would be extremely useful for diagnosis.

## Conclusion

Ultrasound is a very sensitive method, does not present any risks and, in expert hands, allows detecting all the typical signs of PNCS: stenosis of the LRV, congestion of the flow, secondary varicocele and eventual renal failure. In the absence of treatment, stenosis can predispose patients to venous thrombosis with consequent irreversible kidney damage. Failure to diagnose and treat this condition could therefore have serious health consequences for patients.

## Data Availability

All data generated or analyzed during this study are included in this published article and its additional files.

## References

[1] Cohen CT, Kirk S, Desai SB, et al. Diagnosis, clinical characteristics, and treatment modalities of adolescent May-Thurner syndrome-associated deep venous thrombosis. PediatrHematolOncol. 2020. 10.1097/mph.0000000000001968. 33093351

[2] Almohamad FA, Alhimyar M, Esmaeel R, et al. A case report combining Dunbar syndrome and pancreatic neuroendocrine tumor. Ann Med Surg (Lond). 2020;58:91–4. 10.1016/j.amsu.2020.08.038 PMC7486424 32953106

[3] Fiori L, Serrao A, Ferretti A, Chistolini A. The treatment of upper extremities deep vein thrombosis related to thoracic outlet syndrome with direct oral anticoagulants. Phlebology. 2020. 10.1177/0268355520967519. 33106100

[4] Al Faqeeh AA, Syed MK, Ammar M, et al. Wilkie’s syndrome as a rare cause of duodenal obstruction: perspicacity is in the radiological details. Cureus. 2020;12(9):e10467. 10.7759/cureus.10467 PMC7566986 33083170

[5] Yun SJ, Lee JM, Nam DH, et al. Discriminating renal nutcracker syndrome from asymptomatic nutcracker phenomenon using multidetector computed tomography. AbdomRadiol (NY). 2016;41:1580–8. 10.1007/s00261-016-0717-8 27221972

[6] Butros SR, Liu R, Oliveira GR, et al. Venous compression syndromes: clinical features, imaging findings and management. Br J Radiol. 2013;86:20130284. 10.1259/bjr.20130284 PMC3798333 23908347

[7] Singla RK, Sharma T, Gupta R. Retro-aortic left renal vein with left suprarenal vein draining into inferior vena cava. Int J AnatVar. 2010;3:134–7.

[8] Rassi I, Khabbaz Z, Chelala D, et al. A new variant of the posterior nutcracker phenomenon. J VascSurg. 2010;51(5):1279. 10.1016/j.jvs.2009.03.052 19540704

[9] Puig S, Stühlinger HG, Domanovits H, et al. Posterior “Nutcracker” phenomenon in a patient with abdominal aortic aneurysm. EurRadiol. 2002;12(Suppl 3):S133–5. 10.1007/s00330-002-1523-y 12522623

[10] Lamba R, Tanner D, Sekhon S, et al. Multidetector CT of vascular compression syndromes in the abdomen and pelvis. Radiographics. 2014;34:93–115. 10.1148/rg.341125010 24428284

[11] Gozzo C, Giambelluca D, Cannella R, et al. CT imaging findings of abdominopelvic vascular compression syndromes: what the radiologist needs to know. Insights Imaging. 2020;11(1):48. 10.1186/s13244-020-00852-z PMC7078419 32185572

[12] Ananthan K, Onida S, Davies AH. Nutcracker syndrome: an update on current diagnostic criteria and management guidelines. Eur J VascEndovascSurg. 2017;53:886–94. 10.1016/j.ejvs.2017.02.015 28356209

[13] Farina R, Iannace FA, Foti PV, et al. A case of nutcracker syndrome combined with Wilkie syndrome with unusual clinical presentation. Am J Case Rep. 2020;21:e922715. 10.12659/AJCR.922715 PMC7193224 32317620

[14] Marone EM, Psacharopulo D, Kahlberg A, et al. Surgical treatment of posterior nutcracker syndrome. J VascSurg. 2011;54(3):844–7. 10.1016/j.jvs.2011.01.038 21458199

[15] Erben Y, Gloviczki P, Kalra M, et al. Treatment of nutcracker syndrome with open and endovascular interventions. J VascSurg Venous LymphatDisord. 2015;3:389–96. 10.1016/j.jvsv.2015.04.003 26992616

[16] Granata A, Clementi A, Floccari F, et al. An unusual case of posterior nutcracker syndrome. ClinExpNephrol. 2014;18(4):670–1. 10.1007/s10157-014-0932-y 24481951

[17] Labropoulos N, Borge M, Pierce K, et al. Criteria for defining significant central vein stenosis with duplex ultrasound. J VascSurg. 2007;46(1):101–7. 10.1016/j.jvs.2007.02.062 17540535

[18] Belczak SQ, Coelho Neto F, de Araújo WJB, et al. Endovascular treatment of anterior nutcracker syndrome and pelvic varices in a patient with an anterior and a posterior renal vein. BMJ Case Rep. 2020;13(9):e235284. 10.1136/bcr-2020-235284 PMC7478003 32900725

